# To Cultivate Creativity and a Maker Mindset Through an Internet-of-Things Programming Course

**DOI:** 10.3389/fpsyg.2020.01572

**Published:** 2020-07-06

**Authors:** Yu-Lin Jeng, Chin-Feng Lai, Sheng-Bo Huang, Po-Sheng Chiu, Hua-Xu Zhong

**Affiliations:** ^1^Department of Information Management, Southern Taiwan University of Science and Technology, Tainan, Taiwan; ^2^Department of Engineering Science, National Cheng Kung University, Tainan, Taiwan; ^3^Department of E-learning Design and Management, National Chiayi University, Chiayi City, Taiwan

**Keywords:** creatively, peer learning, computational thinking, maker education, learning motivation, World Café model, brainstorm

## Abstract

With the swift development of technology in recent years, entrepreneurs are facing rapid changes in industry. To cope with such changes at home and abroad, The Ministry of Education is actively promoting innovative education with the aim of cultivating students’ entrepreneurship. On this basis, this study proposes an innovative curriculum design based on an Internet-of-Things (IoT) programming course. The reason is that it develops computational thinking skills while students are learning programming and also cultivates logical thinking skills and problem-solving skills, which are critical to entrepreneurship. We also design a number of learning activities that enable students to express their opinions and ideas while gaining more knowledge through peer interaction and discussion. Overall, this study explores the impact of “maker education” on students’ attitudes toward computer thinking. The results indicate that maker education has a positive impact on their ability to learn computer skills. In terms of learning motivation, students are not motivated by maker education and reduce their confidence on the curriculum. The reason may be that the curriculum requires the acquisition of software and hardware skills, which will increase the student’s learning burden, so they more likely to encounter learning disabilities.

## Introduction

Through technical development, industry is changing very rapidly, with many different new ones appearing in recent years. Entrepreneurship has therefore been recognized as a key factor affecting economic development, because entrepreneurs can create and catalyze the necessary structural changes through their entrepreneurship (Hockerts and Wüstenhagen, 2010; Szerb et al., 2019; Horne et al., 2020). Besides, according to Sheshadri et al., 2018, the ability to create novelty is an important root cause of economic growth, and how to foster innovation and maintain global competition is crucial for any company or organization. As a result, the maker movement has received more and more attention because it not only stimulates manufacturers to innovate continuously, but also creates jobs and injects new vitality into urban manufacturing clusters (Wolf-Powers et al., 2017). In addition, the success stories of maker continue to emerge, bringing many innovations and inventions, it also promotes industry innovation and economic development, the purpose is to hope to stimulate entrepreneurship rates and investment (Browder et al., 2019; Holman, 2015). At home and abroad, The Ministry of Education is actively promoting the establishment of maker courses, with the aim of cultivating students’ innovative ability to cope with changes in industry structure. Therefore, governments in recent years have been actively promoting maker education and encouraging the inclusion of maker education in the curriculum of education systems (Huang et al., 2019). In addition, research indicates that maker practices are inextricably linked to the internet and information technology, because some manufacturers need to connect devices that use sensors and telemetry technologies to achieve multiple applications in order to create innovative new products (Sheshadri et al., 2018).

However, it is important to develop students’ basic information skills and computer science skills, as the maker movement is closely related to new technologies and digital tools (Dougherty, 2012). On the other hand, researchers have also expanded the important field of computer science and proposed the concept of computational thinking. The researchers point out that computational thinking is crucial for human because it is not only a problem-solving skill through computer science, but also strategic and effective in solving problems and organizing information (Wing, 2006; Google, 2015). Therefore, computational thinking is an essential skill, but it is difficult to develop students’ computational thinking skills in the current teaching environment. Some researchers point out that due to the pressure of a course’s progress, it is difficult for a teacher to spend more time waiting for the students to fully understand the course. Thus, the teacher will continue to teach, which also makes it difficult for the teacher to grasp the student’s learning situation (Hsu, 2018; Rahmat et al., 2012).

To sum up, the industrial structure is changing rapidly, and we need to cultivate students’ innovative ability and creativity to cope with changes in it and cultivate their entrepreneurship. However, the maker movement has become a key factor influencing entrepreneurship, because maker can not only stimulate manufacturers to innovate continuously, but also promote economic development. On the other hand, there is an inextricable relationship between a maker movement and information technology in order to connect to various sensor or telemetry technologies so that a variety of applications can be implemented through the internet and information technology to create new products (Sheshadri et al., 2018). Therefore, disciplines related to information technology have become important knowledge sources, but cultivating talent is not easy. The main reason is that due to the pressure of course progress, teachers do not have enough time to wait for students to fully understand, thus preventing students from effectively learning professional skills. So, this study proposes an innovative curriculum design based on an Internet-of-Things (IoT) programming course, which integrates maker education teaching strategies designed to allow students to practice programming to cultivate their computational thinking ability. Some researchers also point out the importance of practice, and that teachers should develop students’ professional knowledge and skills in learning practice (Grossman and McDonald, 2008). In addition, there are many software and hardware elements in IoT courses, and students can use their creativity and innovation to build different combinations. On the other hand, this study integrates the peer learning theory to stimulate their brainstorming and achieve deep learning results. Researchers also point out that there is no teacher role in peer learning environments, and students need to help each other to complete learning tasks, so that they can learn different knowledge from peers (Topping, 2005; Chen et al., 2012; Wang, 2016; Shih et al., 2018). According to the Razak and See, 2010, level of motivation has an impact on participation in learning activities, and the study of integrated online peer learning activities to promote students engage in learning, and the results indicated that it has positive impact on improving motivation. Therefore, peer learning is a crucial factor, which not only affects students’ participation in learning activities, but also improves their motivation. However, in order to enhance interaction with peers, we adopt the World Cafe model as a discussion strategy where students can easily exchange ideas with each other. It focuses on strategies and ensures that everyone has equal representation. Students can receive different student opinions and try to solve problems. Finally, in order to explore students’ learning status, this study analyzes learning outcomes, learning motivation and background knowledge, and computational thinking. Overall, there are two hypothesizes in this study as below.

(1) Does maker education enhance students’ computational thinking ability?
(2) Does maker education enhance students’ learning motivation on the programming course?

## Literature Review

### Maker Education

The maker movement has received wider attention in recent years, which has become a way of expressing creativity, and it also encourages students to innovate and increase their entrepreneurship (Halverson and Sheridan, 2014; Hsu et al., 2017). The maker movement is broadly defined as people who engage in the creative production of artifacts, and its main concept is focusing on “do it yourself (DIY)” in one’s daily life (Halverson and Sheridan, 2014; Huang et al., 2019). According to Martin (2015), engaging in activities can facilitate the interest of students, while enabling them to use knowledge and skills to solve problems and thus learn by realizing goals. However, maker education is different from traditional learning. The literature has mentioned that maker education is a new type of education model that takes the student as the center and transforms students’ passive learning into active exploration activities. However, students’ creative thinking skills can be cultivated through maker practices (Niu et al., 2017; Godhe et al., 2019). There are some research results showing that maker education has the advantages of openness, compatibility, sharing, and practicality. It also helps to cultivate students’ creative ability (Yang et al., 2019).

Maker education is a new type of teaching strategy that focuses on learning by doing and provides many exercises to stimulate students’ creativity and innovation ability. However, a maker learning environment can help students use their knowledge to explore and solve problems. Therefore, this research adopts an innovative course design based on the Internet of Things (IoT), which aims to provide students with a large amount of practical experience and learning opportunities. At the same time, they can use software and hardware to implement their different ideas. Involving students in maker learning has a positive impact on learning. Similarly, Baleshta et al. (2015) set up a learning activity that enables students to participate in a design loop using 3D printers. The results show that they enjoy the experience and feel satisfied while growing from the learning activities.

### Computational Thinking

With the development of technology, many researchers have paid greater attention to computational thinking (CT). Wing (2006) points out that CT is one way to solve problems, design systems, and understand human behavior through computer science. In addition, researchers have suggested that people should have computational thinking to cope with technological trends in the digital era. However, part of computational thinking also involves the requirements of computer programming skills, and so there are many educators developing CT concepts through programming (Hambrusch et al., 2009; Israel et al., 2015). Computational thinking is an essential skill in today’s society, so scholars Pinto-Llorente et al. (2016) encourages the development of this skill in different disciplines. On the other hand, the research results indicate that programming has a positive impact at cultivating students’ computational thinking (Pinto-Llorente et al., 2016). In addition to education, Google also emphasizes computational thinking, which can be used in life outside the classroom. Google has developed a series of materials on computational thinking, while also defining four main characteristics of it: pattern recognition, abstraction, algorithm design, decomposition, etc. (Google, 2015). However, there are many different definitions of computational thinking, therefore, integrate some researches Wing, 2006; Barr and Stephenson, 2011; Selby and Woollard, 2013; Google, 2015. Computational thinking includes four main items: (1) abstraction, (2) decomposition, (3) algorithmic thinking, and (4) data representation. These items are designed to help develop strategies and effectively solve problems and organize information.

To sum up, computational thinking is one of the key ways for effective problem-solving through computer science in current society. In order to help students build CT skills, this present study adopts IoT courses, because IoT is a type of programming course. According to Hambrusch et al., 2009; Israel et al., 2015; Pinto-Llorente et al., 2016, use programming is an effective way to cultivate students in computational thinking. We also integrate the Maker strategy, which aims to provide more hands-on opportunities to help them gain deeper learning.

### Peer Learning

Peer learning is an important interaction between peers and can help students establish a learning relationship with their peers. The literature points out in a peer learning environment that most peers are generally of the same class or cohort, and they have a similar situation to each another. In addition, because there is no teacher role, students need to help each other learn, and so they can actively help and support the acquisition of knowledge and skills (Topping, 2005; Wang, 2016). However, researchers (Boud et al., 2014; Meschitti, 2018) mentioned that compared with tradition learning, peer learning helps students achieve better learning outcomes, because it provides more practical opportunities and interaction during the learning process. Therefore, interaction with a peer is a key factor to impact students’ learning outcome. Some researchers (Chen et al., 2012; Shih et al., 2018) note that team members have different levels of background knowledge, and so peers can learn from each other, which facilitates the completion of group tasks. Peer learning also contributes to developing social skills and commutation skills. Lisi (2002) presents that the use of peer learning strategies in schools can improve students’ communication skills by listening to different opinions from peers, so that students can gain a deeper understanding of the subject knowledge.

With these references, one of the most important factors is interaction during peer learning. It not only cultivates students’ social skills and communication skills, but also improves their learning outcomes through a freely communicating discussion environment. On the other hand, peers are made up of students with different levels of knowledge, and so students can hear more opinions and ideas from their peers, which can help them deepen their understanding during learning. Based on those factors, we propose an innovative curriculum design based on an Internet-of-Things (IoT) programming course and integrate peer learning strategies to promote opportunities for students to interact, so that they can help each other and discuss with peers to complete learning tasks. This can help students gain a better understanding of computational thinking.

## Materials and Methods

In this section we summarize the designed learning activities and how to integrate peer learning and maker education into learning activities to cultivate students’ computational thinking skills. We describe data collection and data analysis and divide this section into six phases.

### Participants

This study had a total of 61 students, but 9 students did not fully participate in the learning activities, and so we removed them from the experiment. In total, we had 52 students participating in the study. They were approximately 21 years old and mainly studied at the Department of Information Management. The curriculum integrates software and hardware, which means that students need some relevant knowledge to learn. Therefore, we recruited junior students to conduct this research because they have learned many related computer programming courses, including website programming, object-oriented analysis and design. They have certain programming experience and background knowledge.

### Research Design

**FIGURE 1:**
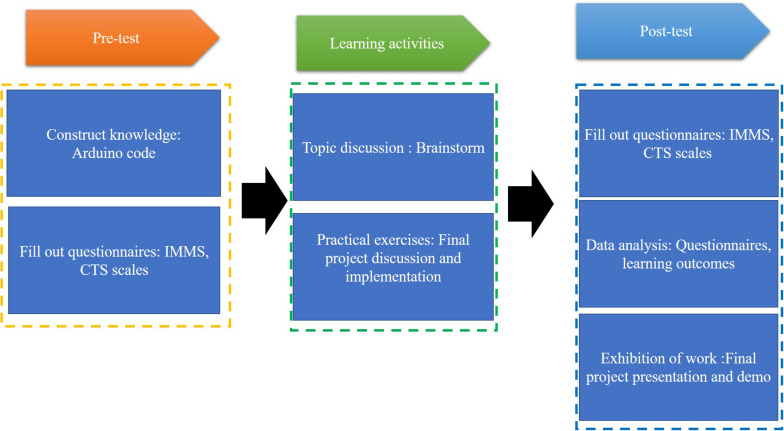
The curriculum processes.

In this study we adopted “Internet-of-Things Mobile Applications Development and Practice” as the research curriculum. The course includes many different sensors and components to enable students use software and hardware to cover many different learning works. In addition, the course is defined as an advanced course and requires a high level of background knowledge, and its operation is slightly more difficult than other programming courses. Therefore, before conducting private learning activities, teachers should focus on developing students’ preliminary background knowledge to avoid their lack of ability to understand and affect learning. Overall, the study consists of three phases (see Figure 1): pre-test, learning activities, post-test.

At the end of the experiment, in order to explore students learning performance, the learning outcomes, learning motivation, computational thinking ability, and background knowledge of the participants were analyzed. In addition, this study integrates peer learning and creative education and aims to help students solve problems together. Students can learn a lot of professional knowledge by discussing and sharing ideas with their peers. The detailed study design is as follows.

In the pre-test stage, teachers focus on the construction of background knowledge, while providing students with an initial understanding and practical opportunities of familiar software and hardware operations. According to some studies (Halverson and Sheridan, 2014; Martin, 2015; Huang et al., 2019), students participating in maker learning activities can help others use knowledge to solve problems while learning by doing. The maker learning environment also provides many practical opportunities that can help develop students’ creative thinking skills through maker practices (Niu et al., 2017; Godhe et al., 2019). On the other hand, students can learn and develop their learning experience by doing projects. Some researchers mentioned that maker education is a learner-centered learning activity, which is beneficial to computational learners to consolidate their understanding and transfer their knowledge and learning from their peers (Bower et al., 2017; Hung et al., 2019). Based on this, we integrated maker education in this study with the aim of providing many practical opportunities to give them a deeper understanding of computational thinking skills. The course includes richness sensors that allow students to use their creativity to implement many different ideas. Finally, before the midterm test, the teachers require students to fill out the Instructional Materials Motivational Survey (IMMS) and perform computational thinking skills (CTS) as the pre-test. The reason is that IMMS is based on the ACRS model. The model uses a variety of factors to explore motivation, so it is suitable for exploring the changes in students’ learning motivation during the experiment. In terms of computational thinking, this study uses the Computational Thinking Scale to measure the level of students’ computational thinking ability, because computational thinking is a method to solve problems, it contains a variety of different thinking skills. The Computational Thinking Scale is a combination of various factors, which is very suitable for exploring students’ computational thinking in experiments.

After being familiarized with the course, for the second phase of the learning activity the teachers focus on student discussions and interactions, with the aim of promoting students’ brainstorming while stimulating their creativity and innovation. Therefore, during the course, the teachers set many topics and issues for students to discuss. On the other hand, the discussion environment integration a “World Café” strategy, a concern for peer interaction, which also emphasizes that everyone must represent their own ideas and express different opinions. Some researchers have integrated the World Cafe method in the learning. The results indicated that World Cafe as a teaching and learning method may provide a lot of help for students engaged in interdisciplinary learning. This is a kind of benefit for participants to reflect and contribute methods (Terry et al., 2015; Estacio and Karic, 2016). In addition, according to studies (Lisi, 2002; Topping, 2005; Boud et al., 2014; Wang, 2016; Meschitti, 2018), a peer learning environment provides a convenient communication environment where students can share ideas and discussions so that they can help and support each other to gain knowledge and skills. It also helps students learn more about knowledge building and achieve a better learning outcome. Based on this, this study uses World Cafe as a discussion strategy. The reason is that the students in each group need to complete their final project, so the group members need a range of viewpoints to solve the problem and achieve the final project. In addition, students are expected to develop their computational thinking skills through the exchange of free ideas.

In the post-test phase, students complete the final project and presentation. The presentation needs to describe how to use sensors to compose a new work and when the work in the presentation needs to be performed. Teachers evaluate students’ problem-solving skills and creativity through group project works. After the final project presentation, teachers require students to fill out the Instructional Materials Motivational Survey (IMMS) and perform computational thinking skills (CTS) as the post-test. Lastly, IMMS, and CTS are analyzed.

### Learning Material

This study applies “Arduino Grove” as the main hardware teaching tools. It contains many elements such as LED, touch sensor, buzzer, light sensor, etc. In the software, we adopt “Android Studio” as the main software development tools. The learning material consists of three parts, including an introduction, practical operation, and a discussion of related issues. In the introductory steps, teachers instruct students on syntax and operation methods, while describing software and hardware concepts to deepen their understanding of the course. Therefore, students need to use software and hardware to complete their learning tasks, while developing their professional skills and computational thinking. Finally, after the introduction and practical steps, the teachers ask different questions based on each group’s lesson, and then the students can use their knowledge to discuss.

### Design Learning Activities With World Café

The literature review of peer learning reveals that it can help students to gain deeper knowledge. On the other hand, students can listen to other opinions and ideas for the completion of group tasks, which also can enhance their learning outcome. Therefore, there are two key factors in peer learning: interaction and communication. To promote student interaction and networking opportunities, this study adopts “World Café” as a method to participate in discussions. The literature has mentioned that World Café can foster constructive dialog, which can help assess collective intelligence and create innovative possibilities. In addition, in the World Café dialog environment, participants can use their wisdom and creativity to explore issues and also enable a group of people to communicate around issues that are important to the entire group (Fallon and Connaughton, 2016; MacFarlane et al., 2017). In short, it is a method of emphasizing dialog, and students have more opportunities to listen and share ideas and opinions with other students. In the World Café learning environment, the students use their wisdom to explore problems in an attempt to solve them, which can help students gain a rich learning experience while deepening their learning. Based on this, we adopt the World Café into learning activities and conduct a three-part process with a total of 52 participants. We randomly divide students into multiple groups, each of which has different learning tasks on topics, and the session topics are based on “IoT.” On average there are 5–6 people sitting down around the table in a group. There are three session rounds. Each session lasts about 20 min. In addition, according to the Schieffer et al. (2004) and World Café (2015) guideline, there are three roles, “Table host,” “Participant,” and “Café host,” described as follows.

Table host: welcomes travelers from other tables and share key insights briefly to facilitate connection ideas, while encouraging them to talk.
Participants: contribute opinions and carry forth key ideas or themes, while peers also need to listen to the ideas of the participants.
Café host: welcomes participants to enter and explain the process as well as the spirit and purpose of the event.

Before the session, the Café host roughly guided the process for the participants. At the beginning of the session, the table host briefly introduced the topic and asked participants to spend 1–2 min to think about the topic direction and understand the topic, while they have 1 min to write down their ideas. Subsequently, the table host asked participants who spoke in sequence at each table for 2 min to share their ideas (10–12 min in total, including a 1-minute buffer time for members to think and understand the discussion). As they began the World Café session, participants were encouraged to pick up pens to write individual ideas or share memos on prepared A1-size paper so that people can more easily grasp the topics and content in the next round.

**FIGURE 2:**
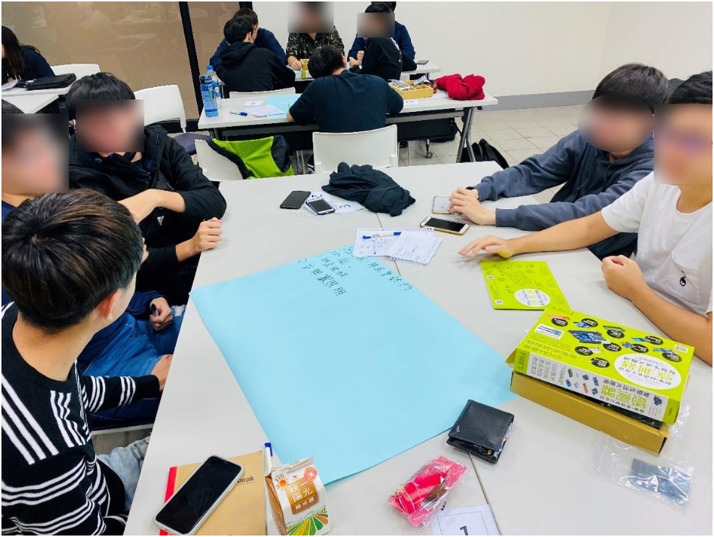
Write individual ideas or shared memos.

**FIGURE 3:**
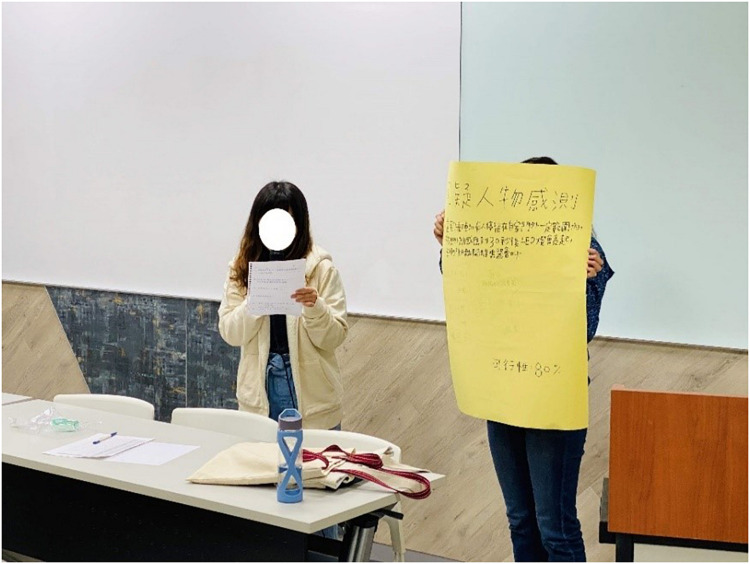
Present the results of the conversation to classmates.

The process is shown below in Figure 2. After the first round, all members except the table host moved to another table. The table host summarized the previous dialog results and explained the topic to the new members in 1 to 3 min; subsequently, the new members had a conversation on the topic. After the end of the three-round session, each table host silently thinks about the results of the three rounds of sessions and writes them down. Finally, the Café host asked each group of table hosts to present summary conversation results and topics to classmates in 5 min. The presentation is shown in Figure 3.

### Questionnaire Items for Participants’ Learning Motivation

To measure student learning motivation, this study adopted the Instructional Materials Motivational Survey (IMMS). IMMS is based on the ARCS motivation model and consists of four factors, including Attention, Relevance, Confidence, and Satisfaction (Keller, 1983; Huang et al., 2006). Based on the literatures (Liao and Wang, 2008; Bolliger et al., 2010; Huang et al., 2010; Julià and Antolí, 2019), the description of the ACRS model factors are summarized as follows.

(1) Attention: attracting learners’ attention can increase learning interest and enhance their willingness to learn.
(2) Relevance: provide relevant learning materials or meet their needs for learners, thus making them more concerned or willing to learn in class.
(3) Confidence: teachers should help students build positive expectations, which will help them feel more confident about success and completing learning tasks.
(4) Satisfaction: satisfying learners during the learning process has a positive impact on learners and allows them to continue learning.

To sum up, we adopt IMMS to measure student learning motivation. The IMMS scale is based on the 5-point Likert scale, with 5 points indicating “strongly agree” and 1 point indicating “strongly disagree.” In addition, the scales’ reliability coefficient score ranging from 35 to 180 is 0.96 (Keller, 2006, unpublished).

### Questionnaire Items for Participants’ Computational Thinking

In terms of computational thinking, we adopt the computational thinking scale (CTS), developed by Korkmaz et al. (2017). CTS consists of five factors: creativity, cooperation, algorithmic thinking, critical thinking, and problem-solving, for a total of 29 items. (1) creativity: this is not a way of thinking alone, including critical thinking and problem-solving. It helps students discover their creativity and method for solutions (Korkmaz et al., 2017). (2) cooperation: cooperative learning requires teamwork and effective communication, and so students need to help each other to achieve their learning goals (Nam, 2014; Altun, 2017). (3) algorithmic thinking: “algorithms are central to both computer science and computational thinking.” They are the basis of tasks that everyone engages in and present precise steps to solve problems (Yadav et al., 2017). (4) critical thinking: it is an important skill for computing. To solve a problem, students need knowledge and skills to evaluate the problem and generate a solution (Ater-Kranov et al., 2010). (5) problem-solving: it is an important skill and covers strategic problem-solving and effective problem-solving (Korkmaz et al., 2017).

To sum up, we adopt CTS to measure student computational thinking. The CTS scale is based on the 5-point Likert scale, with 5 points indicating “strongly agree” and 1 point indicating “strongly disagree.” In addition, the Cronbach alpha consistency coefficient calculated for the CTS scale is 0.822 (Korkmaz et al., 2017).

## Results and Discussion

In order to further explore the learning performance of students, we use the final project as the capstone course to evaluate their comprehensive skills and learning status. According to Laplante et al. (2019), capstone courses intend to provide students with a meaningful experience and provide them with a productive environment to achieve learning outcomes, so that they can use their learning knowledge. On the teacher side, teachers can use the students’ learning outcomes to assess their learning situation. However, in order to effectively collect data, we must ensure that participants first complete their final project and then complete the IMMS and CTS scale.

### Research Hypothesis 1: Does Maker Education Enhance Students’ Computational Thinking Ability

In order to evaluate the impact of “Maker Education” on participants’ computational thinking ability, we use pre-test and post-test methods to measure their various computational thinking abilities. The reason is that after the midterm exam, the course focuses on students, and integrating the educational theories of maker education gives them many opportunities for practice. Therefore, we collect data during the midterm and final exams as the pre-test and post-test. Finally, we use paired sample t-tests to analyze whether participants’ means differ significantly. In the CTS scale, the mean of all dimensions is between 3 and 4. There are also significant differences in four dimensions, including “Creativity,” “Algorithmic thinking,” “Cooperativity,” and “Critical thinking” (for pre-test and pro-test scores, see Table 1). In the pre-test stage, the average score is 3.43, and the range score is 3.13–3.68, indicating that students have a certain level of computational thinking ability. After the post-test, the average score is 3.518 and the range score is 3.26–3.76. Compared with the pre-test, maker education teaching improves students’ ability at computational thinking.

**TABLE 1:** CTS analysis results.

Participant	Dimensions	Pre-test		Post-test		*t*	*p*
		M	SD	M	SD		
Students (*n* = 52)	Creativity	3.60	0.38	3.71	0.47	−2.18	0.03*
	Algorithmic thinking	3.13	0.68	3.26	0.67	−2.31	0.03*
	Cooperativity	3.68	0.71	3.76	0.51	−0.98	0.33
	Critical thinking	3.40	0.56	3.53	0.56	−2.21	0.03*
	Problem solving	3.32	0.47	3.33	0.46	−0.11	0.91

*p < 0.05.

### Research Hypothesis 2: Does Maker Education Enhance Students’ Learning Motivation on the Programming Course

In order to further explore participants’ attitudes toward maker education, we use IMMS to analyze their learning motivations (for pre-test and post-test scores, see Table 2). In the pre-test, the average score is 3.69, with a range of 3.44–3.86, which means that students have a certain motivation for learning the programming course. In the post-test stage, the average score is 3.70, and the range is from 3.33 to 3.91, which is a slight improvement overall. However, further research on each dimension indicates that results are enhanced in two dimensions, including attention and satisfaction. Especially in terms of satisfaction, participants are significantly different. The satisfaction score is between 3.75 and 3.91, which means that most participants generally believe that the curriculum is integrated with the maker education, which helps the participants attain a sense of accomplishment. On the other hand, in terms of confidence, it has dropped significantly, with confidence scores ranging from 3.44 to 3.33. This means that maker education cannot effectively build confidence and actually has the opposite effect during the programming course.

**TABLE 2:** IMMS analysis results.

Participant	Dimensions	Pre-test		Post-test		*t*	*p*
		M	SD	M	SD		
Students (*n* = 52)	Attention	3.71	0.52	3.75	0.51	−0.71	0.48
	Relevance	3.86	0.49	3.84	0.41	0.45	0.65
	Confidence	3.44	0.40	3.33	0.47	2.20	0.02*
	Satisfaction	3.75	0.56	3.91	0.60	−2.11	0.04*

*p < 0.05.

### Maker Education Can Promote the Development of Computational Thinking

According to Table 1, students’ computational thinking ability achieves a certain positive impact. This indicate in the maker learning environment that most students have mastered a certain degree of computational thinking ability. In addition, there are significant differences in four dimensions, including “creativity,” “algorithmic thinking,” “cooperativity,” and “critical thinking,” whereas only “problem-solving” has a slight increase.

One reason could be that the course requires high thinking ability and sufficient background knowledge, but the key ability of students to understand the problem is not stable. Therefore, even if students have a lot of ideas on the problem, they will not necessarily grasp the key point to the problem. In order to improve students’ problem-solving skills, some scholars point out using heuristics to solve problems has a positive impact. In addition, effective problem-solving experience may motivate students to solve successful problems, which may also have a positive impact on their skills and help them expand their thinking (Karatas and Baki, 2017). Based on this, in future teaching, teachers can strengthen the construction of basic concepts through basic example exercises. Students can thus gain more practical opportunities and gain more problem-solving experience. After they understand more stable concepts, the teachers can carry out more in-depth teaching.

### Maker Education Does Not Effectively Improve Students’ Motivation to Learn in Programming Courses

According to the results in Table 2, there is only one significant difference in student satisfaction. This indicates that students are satisfied with the learning activities. Students need to use their ideas and knowledge to implement and demonstrate the final project. Thus, the learning results of each group are different, and they can build their own sense of accomplishment through maker education, allowing them to be satisfied with the learning. However, in terms of confidence, it has dropped significantly, which means that maker education for the programming course does not actually build student confidence. It is speculated that students need to learn both hardware and software knowledge, which can put a heavier burden on their learning, and so they are more likely to encounter learning disabilities. In addition, as the learning burden increases, it also affects their cognitive load.

With regard to the cognitive load theory, Sweller et al. (1998) and Sweller (2011) point out that when dealing with novel information, human cognition requires a large amount of information storage. However, the amount of novel information that can be processed at any given time is very limited, and so it is difficult to add information without sufficient working storage capacity. This means that course design and learning material design bring forth more learning load to students, which can affect their learning. Therefore, how to effectively reduce the cognitive load of students is an important issue in learning. According to the report by Küçük et al. (2014), students have lower cognitive load and exhibit more positive attitudes. On the other hand, Cheng (2017) states that students have a smaller cognitive load and may have stronger motivation and a more positive attitude, which also increase their participation in learning activities. In addition, the scholar Cheng (2017) suggests that solving problems or giving guidance with prompts has a positive effect on building learner confidence. Based on these findings, teachers can use assistive tools to guide students’ learning while reducing their cognitive load and helping them build learning confidence. As Henry and Marrs (2015) noted, students learn a language very irregularly due to a lack of motivation and confidence. Therefore, scholars suggest that daily task-based online social networking (DOTS) can be applied to learning, because it has a positive impact on improving students’ learning confidence and learning motivation.

## Conclusion

In recent years, the maker movement has received more and more attention because it not only stimulates manufacturers to innovate continuously, but also promotes the emergence of new companies and economic development. The government actively promotes “Maker Education” to develop students’ innovative ability to cope with the rapidly developing industry. This present study proposes an innovative curriculum design based on an Internet-of-Things (IoT) programming course. The course integrates peer learning strategies by encouraging students to stimulate their creativity and innovation during discussions. On the other hand, it trains students’ entrepreneurship by integrating maker education into a programming course and develops their computational thinking ability through the course. However, in order to encourage interaction between students, this course integrates the “World Cafe” strategy into the learning activities. After the experiment, we collected the data and analyzed it and discussed participants’ learning motivation and computational thinking. Overall, two main results are derived from the analysis of the two experimental hypotheses.

(1) Maker education has a positive impact on computational thinking:

After the experiment, according to Table 1, students have a positive influence on computational thinking. In addition, in order to further explore the factors of CTS, it is shown that the experiment has significant differences in improving students’ “creativity,” “algorithmic thinking,” and “critical thinking,” but it has increased slightly in terms of problem-solving factors. Overall, this study proposes an innovative curriculum design aimed at improving students’ thinking ability in computing.

(2) Maker education does not effectively enhance students’ learning motivation:

As can be seen from Table 2, in terms of satisfaction factors, there are significantly different improvements. Overall, this study did not effectively improve students’ learning motivation. In particular, this experiment will have a negative impact on the confidence factor. It is speculated that students need to learn both hardware and software knowledge, which can put a heavier burden on their learning, and so they are more likely to encounter learning disabilities.

However, in terms of confidence, the experiment may be adding prompting mechanisms to guide students’ learning, with the aim of building students’ confidence in learning with appropriate help, while reducing barriers to learning, thereby leading to a reduction in learners’ confidence. Some researcher believe that giving guidance or assistive tools can not only build students’ confidence in learning, but also increase students’ participation in learning activities (Cheng, 2017). In addition, in terms of learning activities, it can integrate daily online social networks based on tasks to cultivate regular learning, which can not only improve students’ learning confidence, but also have a positive impact on learning motivation (Henry and Marrs, 2015).

## Data Availability Statement

The original contributions presented in the study are included in the article, further inquiries can be directed to the corresponding author.

## Ethics Statement

Ethical review and approval was not required for the study on human participants in accordance with the local legislation and institutional requirements. Written informed consent from the participants legal guardian was not required to participate in this study in accordance with the national legislation and the institutional requirements. Written informed consent was obtained from the individuals for the publication of any potentially identifiable images or data included in this article.

## Author Contributions

S-BH was responsible for analyzed data and interpreted data. Y-LJ was responsible for designed research framework and collected data and revised articles. C-FL was responsible for interpreted data and revised articles. P-SC was responsible for collected data and revised articles. H-XZ was responsible for analyzed data and designed research framework. All authors read and approved the final manuscript.

## Conflict of Interest

The authors declare that the research was conducted in the absence of any commercial or financial relationships that could be construed as a potential conflict of interest.
